# Microstructure, Mechanical and Corrosion Properties of Copper-Nickel 90/10 Alloy Produced by CMT-WAAM Method

**DOI:** 10.3390/ma17010050

**Published:** 2023-12-22

**Authors:** Marcin Maleta, Joanna Kulasa, Aleksander Kowalski, Paweł Kwaśniewski, Sonia Boczkal, Marek Nowak

**Affiliations:** 1 Łukasiewicz Research Network—Institute of Non-Ferrous Metals, ul. Sowińskiego 5, 44-100 Gliwice, Poland; 2 Faculty of Non-Ferrous Metals, AGH University of Science and Technology, al. Adama Mickiewicza 30, 30-059 Kraków, Poland

**Keywords:** copper-nickel alloy, WAAM, 3D printing, additive manufacturing, microstructure, mechanical properties, corrosion properties

## Abstract

In the case of copper and its alloys, Wire Arc Additive Manufacturing (WAAM) 3D printing technology is mainly used to produce elements for the maritime industry and research has focused on the use of Cu-Al alloys. There is little information devoted to the use of Cu-Ni alloys in this technology, which are also widely used in the maritime industry. In this work, tests were carried out on the microstructure, mechanical properties, and corrosion properties in a 1M NaCl solution of Cu-Ni 90/10 alloy 3D walls printed using the WAAM method. The obtained objects are characterized by a microstructure with elongated column grains and particles of the Ni-Ti phase, hardness in the range of 138–160 HV10, ultimate tensile strength of 495–520 MPa, yield strength of 342–358 MPa, elongation of 16.6–17.9%, and a low average corrosion rate of 7.4 × 10^−5^ mm/year. The work shows that it is possible to obtain higher mechanical properties of Cu-Ni 90/10 alloy 3D objects produced using the WAAM method compared to cast materials, which opens up the possibility of using this alloy to produce objects with more complex shapes and for use in corrosive working conditions.

## 1. Introduction

Additive manufacturing (AM) technologies are developing dynamically and are becoming increasingly important in industry. Compared to conventional production processes, additive manufacturing offers the possibility of producing elements with more complex shapes. Moreover, in many cases, the use of these technologies proves to be more economically beneficial due to the shorter production chain and less waste material. Among the many available AM technologies, one of the 3D printing methods of dimensional elements is the WAAM technology. In the WAAM technology, a three-dimensional element is produced by depositing successive layers of a wire melted by an electric arc [1]. Compared to 3D printing technologies in which the feedstock material is powder, WAAM technology allows the production of metal elements of large sizes and on a large scale. However, the manufactured elements are characterized by lower accuracy, large dimensional tolerance, and lower surface quality, which requires additional surface treatment stages. Only metallic materials that are characterized by good weldability can be used for WAAM technology. In addition, elements manufactured using this technology are often characterized by high residual stresses and deformations due to the high heat input. Despite its disadvantages, WAAM technology provides the possibility to produce a complex shape in one step, which reduces the need for manual assembly and welding and also shortens the final processing time due to the production of objects with a shape similar to the designed one. This allows the production time in many cases to be significantly shortened [2,3].

Due to the possibility of producing large structural elements using this method, the main group of metallic materials used are steels [4], aluminum alloys [5], titanium alloys [6], and nickel alloys [7]. In the case of copper and its alloys, the WAAM technology is primarily used to produce large elements for the maritime industry. In the literature, many works focus on the production of aluminum bronze elements [8,9,10,11,12], which are used to produce, among others, ship propellers [13]. In addition to the Cu-Al alloys used for WAAM printing, research was conducted on the production of elements from other copper alloys such as Cu-Cr-Zr [14], Cu-Si [15], and Cu-Ni [16].

Among these alloys, copper alloys with the addition of nickel are widely used in the maritime industry and are characterized by very good corrosion resistance. The most common grades of cupronickel are alloys with 10 and 30% nickel content. Cu-Ni alloys form a solid solution and exhibit complete solubility in both the liquid and solid states. The addition of nickel affects the mechanical and physical properties of the alloy. As the nickel content increases, the tensile strength, yield strength, and tensile strength at elevated temperatures and corrosion resistance increase. However, the electrical and thermal conductivity decreases and the elongation decreases. Cu-Ni 90/10 alloys with the addition of manganese and iron are characterized by very good plasticity and are used to produce elements in seawater, oil, and gas transport systems, as well as in shipping. Alloys with a higher nickel content are used in the maritime industry, which requires higher mechanical properties and even higher resistance to seawater flow and sand abrasion. The addition of iron to the Cu-Ni alloys increases corrosion resistance and slightly mechanical properties, while manganese improves casting properties; binds sulfur, which is unfavorable for hot processing; and also improves strength. An important addition, especially for Cu-Ni alloys intended for welding, is the addition of titanium, which allows the creation of pore-free welds due to the high affinity of this element to atmospheric gases [17]. 

Cu-Ni 90/10 alloys produced by conventional production techniques in the casting and plastic forming process have been the subject of many studies of mechanical properties [18,19,20] and corrosion properties in the conditions of seawater and other solutions [21,22,23,24,25,26,27,28]. The microstructure and properties of the friction-welded CuNi10 alloy were also investigated [29]. The Cu-Ni 90/10 alloy is also used to create anti-corrosion coatings on steel materials, which, similarly to the epoxy polymer corrosion inhibitors used [30], are intended to limit the corrosion rate of less resistant steel. The authors of the work [31] demonstrated an increase in the effectiveness of protection against corrosion of mild steel by approximately 92% by electrodeposition of a Cu-Ni 90/10 copper alloy coating on its surface.

Despite the rapidly developing knowledge, there is little information in the literature on WAAM about the use of copper-nickel alloys in this technology. The authors of [32] made the first attempts to produce three-dimensional elements from the Cu-Ni 70/30 alloy using the WAAM technology and, in their article, they provide preliminary theoretical and experimental guidelines for future research focusing on the use of Cu-Ni alloys in the WAAM technology. This article adds to the still little information in the field of manufacturing spatial objects by the WAAM method from copper-nickel alloys. The article presents the results of microstructure, mechanical properties, and electrochemical tests of a copper-nickel alloy with a Ni content of 10%, produced using the WAAM 3D printing method. The article shows that it is possible to obtain three-dimensional objects from the Cu-Ni 90/10 alloy with higher strength properties compared to the cast elements, which opens the possibility of using this alloy to produce objects by the WAAM 3D printing with more complex shapes intended for work in corrosive environments.

## 2. Materials and Methods

The test material was WAAM 3D printed walls with dimensions of 37 mm × 76 mm × 7 mm. A commercial welding wire with a diameter of 1 mm made of a Cu-Ni 90/10 alloy was used for printing. The chemical composition of the wire is presented in Table 1.

**Figure 1:**
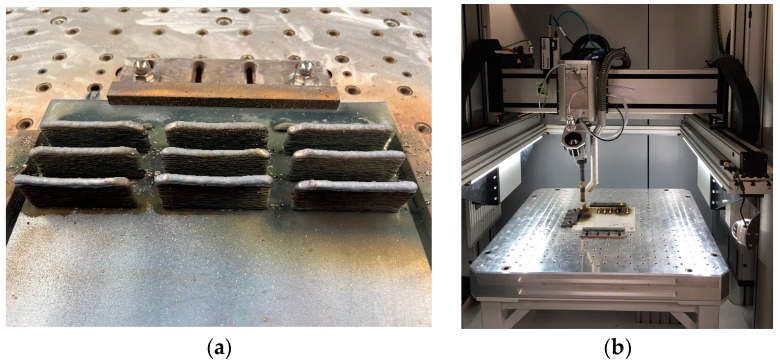
Attempts to produce three-dimensional elements from the Cu-Ni 90/10 alloy using the CMT-WAAM method: (**a**) the printed batch of three-dimensional elements and (**b**) the printer working chamber.

The printing process was conducted on a GEFERTEC WAAM 3D printer equipped with a CMT (Cold Metal Transfer) MIG (Metal Inert Gas) welding machine from Fronius. The initial process parameters were selected based on first-hand experience after trying to print with other copper alloys. The parameters were selected until the metal splash effect was eliminated and regular shapes were obtained. After creating an initial series of walls and selecting initial parameters, a pilot batch of spatial elements for testing was manufactured (Figure 1).

The walls were printed on a S235 steel substrate. An Ar/He 50/50 gas mixture was used to create a protective atmosphere for the electric arc. The torch stroke was 1.2 mm, the torch speed was 800 mm/min, and the control temperature measured after applying each subsequent layer was 150 °C. The voltage and current parameters used for printing elements are presented in Table 2. 

**Figure 2:**
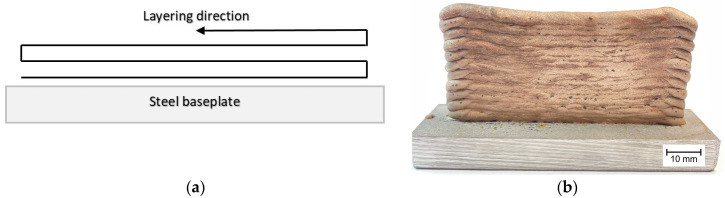
Printed 3D wall for testing: (**a**) direction of layer deposition and (**b**) the wall after the surface cleaning process.

Spatial objects in the form of a single-layer wall were printed using the layer-on-layer technique (Figure 2). The manufactured three-dimensional elements were subjected to macrostructure, microstructure, hardness, static tensile, and corrosion tests. The macrostructure and microstructure were examined in transverse and longitudinal sections to the printing direction. After the grinding and polishing process, the samples for macrostructure testing were etched in an aqueous solution of HNO_3_ acid and for microstructure testing, they were etched in an aqueous FeCl_3_ solution. Macrostructure observations were made using a Keyence VHX7100 digital microscope (Keyence, Osaka, Japan) and microstructure observations were made using an Olympus GX71 light microscope (LM) (Olympus, Tokyo, Japan), a Zeiss LEO scanning electron microscope (SEM) (Zeiss, Jena, Germany), and a Tecnai G2 transmission electron microscope (TEM) (FEI, Hillsboro, OR, USA) equipped with an electron beam scanning module (STEM). The chemical composition in micro areas was examined using an energy dispersive (EDS) detector. Microstructure SEM observations and chemical composition measurements using an EDS detector were carried out with an electron high tension (EHT) of 20 kV and in the secondary electron (SE) mode. Samples for transmission electron microscopy testing were ground and then subjected to ion beam milling to prepare a thin film using a Leica EM RES101 beam milling device (Leica, Wetzlar, Germany). Hardness was measured using the Vickers method. The tests were performed with the indenter load of 10 kg for 15 s. Three indentations were made in 5 areas of the transverse and longitudinal section of the produced walls, based on which the average value for individual areas was calculated.

For the tensile testing process, samples were taken from the transverse and longitudinal section of the produced walls. To prepare samples for the longitudinal tensile test, an additional wall with dimensions of 76 mm × 76 mm × 7 mm was manufactured. Three tensile tests were performed for each cross-section at a speed of 2 mm/min. The values of the ultimate tensile strength (UTS) and yield strength (YS) were determined. The elongation (A_50_) was calculated based on the dimensional changes plotted on the stretched sample. The static tensile test was carried out on an Instron 100 kN universal testing machine.

To evaluate the corrosion behavior, potentiodynamic tests were carried out after 1 h of sample stabilization in open circuit conditions in a three-electrode system (OCP). The working electrode was the tested sample, the reference electrode was the Ag/AgCl/3M KCl (E = 207 mV) electrode, and the reference electrode was a platinum plate. The tests were carried out in the potential range of ±100 mV, in relation to the OCP potential, with a potential change rate of 0.001 V/s. The tests were conducted in a 1 molar NaCl solution at 25 °C. The average values of the corrosion parameters: current density (I_corr_), corrosion potential (E_corr_), and polarization resistance (R_p_) were determined based on the obtained curves for three measurements and using the Tafel equation. The test was carried out using the Autolab PGSTAT302 device from ECO CHEMIE (Utrecht, The Netherlands). The corrosion rate (mm/year) was calculated using the NOVA software (version 2.1.5.) included in the electrochemical testing station. The formula from the ASTM G59-97 standard [33] was used to determine the corrosion rate.

## 3. Results and Discussion

**Figure 3:**
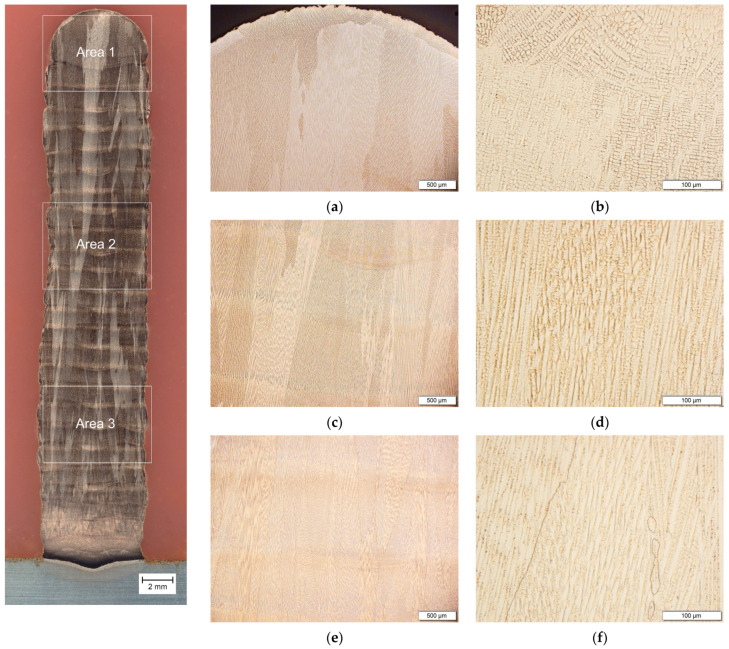
Macrostructure and microstructure (LM) of the Cu-Ni 90/10 alloy 3D wall in transverse cross-section: (**a**,**b**) area 1; (**c**,**d**) area 2; and (**e**,**f**) area 3.

The macrostructure of the produced spatial object in the transverse section is characterized by the presence of large columnar grains (Figure 3) arranged in the direction of heat dissipation to the steel base. Smaller grains are observed near the steel baseplate itself, which is caused by refinement by the iron diffusing from the steel plate and rapid heat dissipation. The transition paths of the subsequent layers are visible but this does not affect the integrity of the material throughout the cross-section. Differences in the size of grains are presented in images of the microstructure from individual areas of the cross-section of the manufactured wall (Figure 3a–f). The grains in the upper part are more than 500 µm wide, while in the lower part they range from 100 to 250 µm. The microstructure of the produced walls is characterized by a dendritic grain morphology with variable orientations.

**Figure 4:**
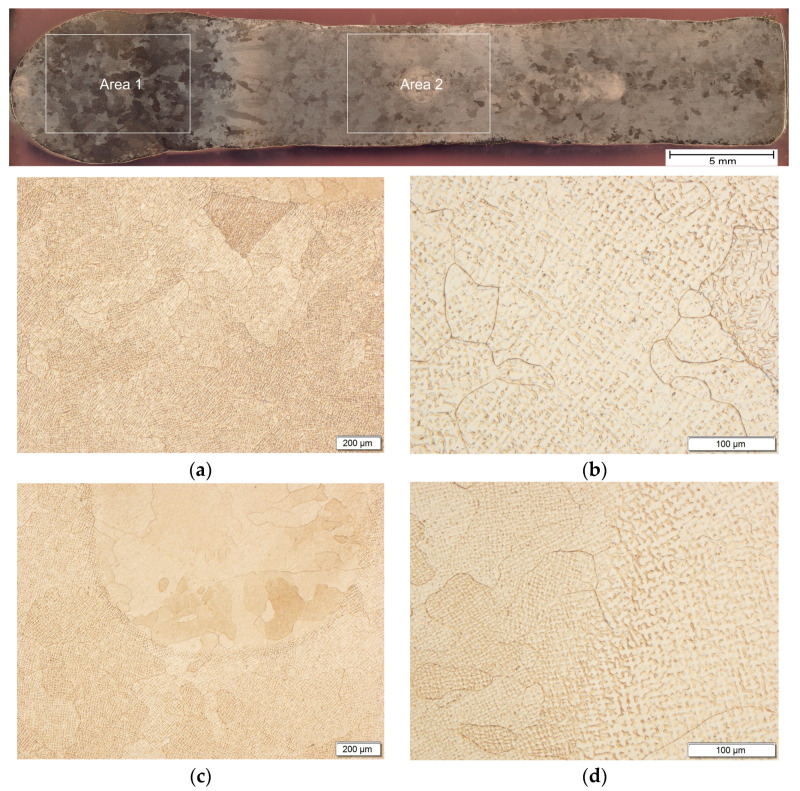
Macrostructure and microstructure (LM) of the Cu-Ni 90/10 alloy 3D wall in the longitudinal cross-section: (**a**,**b**) area 1 and (**c**,**d**) area 2.

The macrostructure in the longitudinal section shows a cross-section of columnar grains oriented towards the baseplate (Figure 4). In the middle of the wall, the occurrence of grains with variable cross-section shapes, ranging from columnar to nearly equiaxed, was observed. Differences in the shape of grains indicate the existence of inhomogeneities in the macrostructure and microstructure. Traces of applying subsequent layers are also visible. In the microstructure of the longitudinal section, grains with a dendritic morphology and shape close to equiaxed were observed, with a predominant size above 500 µm (Figure 4a,c). The occurrence of single grains with an average cross-sectional chord ranging from 35 µm to 250 µm was found (Figure 4b,d).

**Figure 5:**
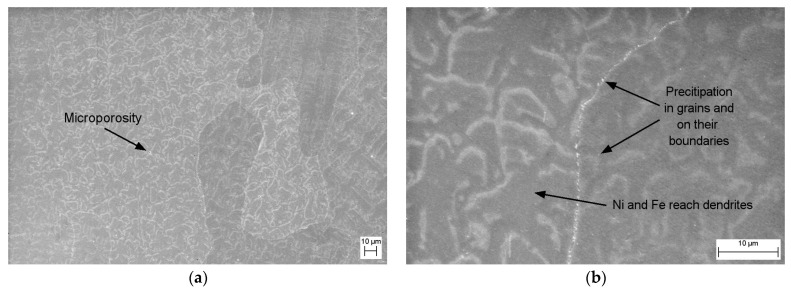
Microstructure (SEM) of the Cu-Ni 90/10 alloy 3D wall in the transverse cross section: (**a**) area 1 and (**b**) area 2.

**Figure 6:**
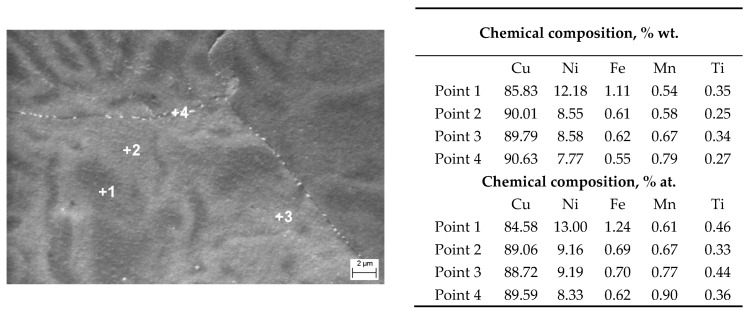
Microstructure (SEM) of the Cu-Ni 90/10 alloy 3D wall in transverse cross-section with the results of EDS point chemical microanalysis.

The SEM-EDS point microanalyses of the chemical composition confirmed the presence of all alloy components in the matrix. In the area of dendrites with a darker shade (Figure 5a,b), the analyses indicated the occurrence of higher Fe and Ni content in relation to the lighter areas of the interdendritic spaces (Figure 6). A small amount of microporosity was located in mainly marginal areas of sample (Figure 5a). Numerous irregularly shaped precipitates were found on the surface of the grains and their boundaries (Figure 5b).

**Figure 7:**
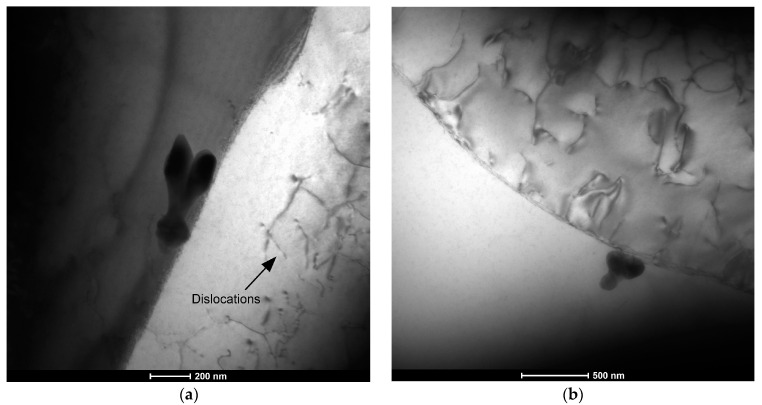
Microstructure (TEM) in the bright field (BF) of the Cu-Ni 90/10 alloy 3D wall: (**a**) area 1 and (**b**) area 2.

The precipitates at the grain boundaries and in the matrix observed using TEM (Figure 7a,b) are characterized by a size of approximately 300 nm. In addition, TEM images show the presence of dislocations, which were observed both in the matrix area (Figure 7a) and in the boundary areas (Figure 7b), which probably affect the hardening of the material.

**Figure 8:**
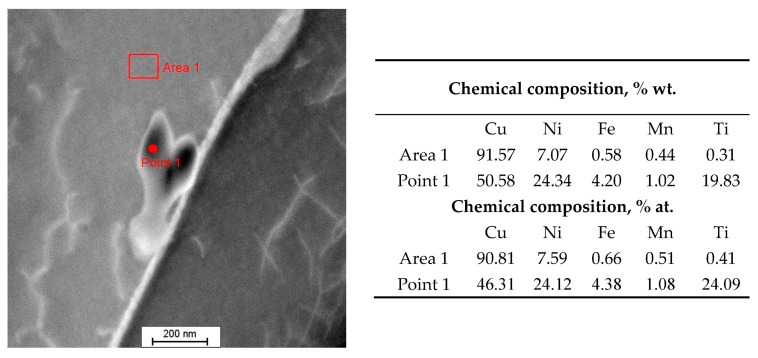
Microstructure (STEM) of the Cu-Ni 90/10 alloy 3D wall with the results of point microanalysis of the chemical composition of the precipitate (EDS).

Microanalyses of the chemical composition in the area of precipitates using the STEM-EDS showed an increased content of alloying elements, especially Ni and Ti (Figure 8).

**Figure 9:**
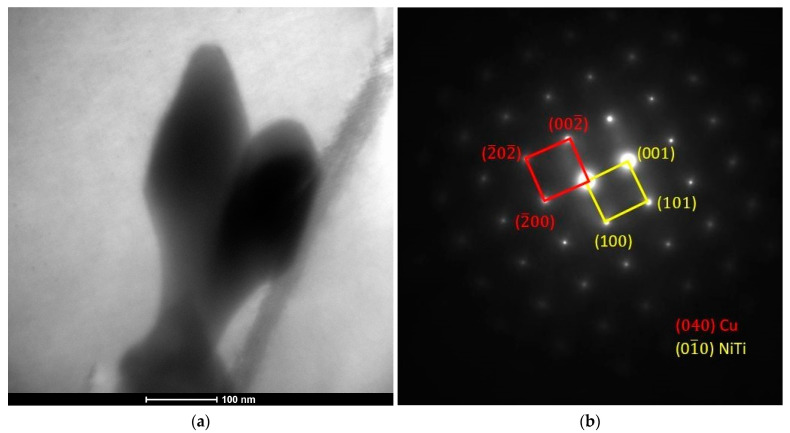
Microstructure (TEM-BF) of the Cu-Ni 90/10 alloy 3D wall: (**a**) Ni-Ti particle on the grain boundaries and (**b**) selected area diffraction (SAD) of the precipitate.

The point diffraction results (Figure 9) and the results of microanalysis of the chemical composition performed using STEM-EDS, which showed the ratio of the percentage of atomic content of Ni:Ti/1:1 (Figure 8) and allowed the identification of the precipitate as a phase with the Ni-Ti formula in the international diffraction database with cubic structure and Pm-3m (221) space group. Titanium added to welding alloys from the Cu-Ni 90/10 group is intended to combine with atmospheric gases and reduce the formation of porosity. The authors of work [32] showed in their research that with the printing parameters used for the Cu-Ni 70/30 alloy with a lower Ti content, TiO_2_ particles are present in the alloy matrix. However, they do not provide information about the protective gas used during the printing process. They also indicate the deoxidizing effect of the Mn and Si elements, the content of which decreased after the printing process. In the tested samples made of the Cu-Ni 90/10 alloy printed in an Ar/He 50/50 gas atmosphere, no decrease in the content of these elements was observed and no TiO_2_ precipitates were observed in the microstructure, only accumulating Ni-Ti phase precipitates both in the area of the grains and in larger amounts at their boundaries were observed.

**Figure 10:**
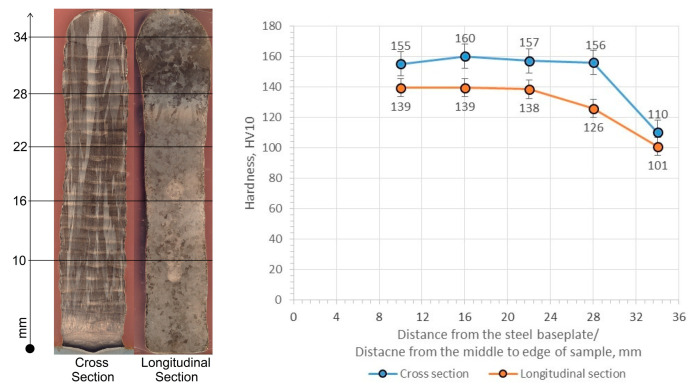
Hardness distribution in the transverse and longitudinal section of the Cu-Ni 90/10 alloy 3D wall.

The hardness of the manufactured wall in the cross-section differs from the hardness in the longitudinal section, which results from the macro- and microstructure. The average hardness in the transverse section ranges from 110 to 160 HV10, while in the longitudinal section it ranges from 101 to 139 HV10 (Figure 10). A clear trend of decreasing hardness in the edge areas of the wall can be seen, which is probably related to the slight microporosity present in those areas or the accumulation of more heat, which annihilates the dislocations present and reduces the hardening effect. Considering the stable hardness measurement results, the average value in the transverse section is 155–160 HV10, while in the longitudinal section it is 138–139 HV10.

**Figure 11:**
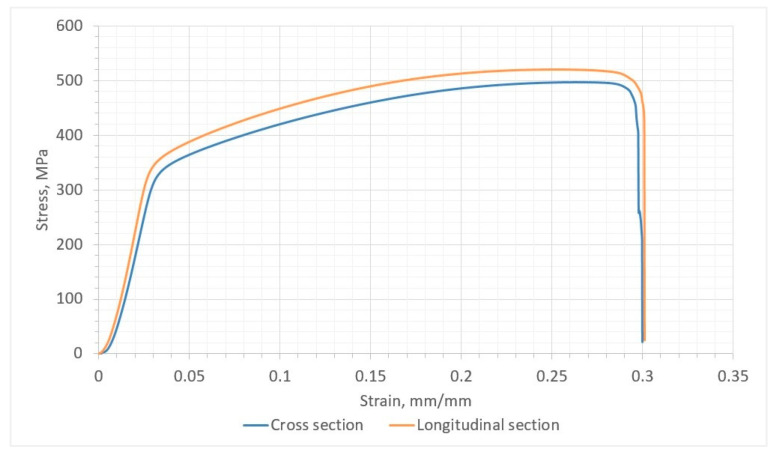
Tensile test curve for samples taken from the transverse and longitudinal section of the Cu-Ni 90/10 alloy 3D wall.

Slight differences in mechanical properties in the transverse and longitudinal sections are also indicated by the results of the static tensile test (Figure 11). For samples taken from the transverse section, the average value of ultimate tensile strength was 495 MPa, the yield strength was 342 MPa, and the elongation was 17.9%, while for the samples taken from the longitudinal section, the average value of the ultimate tensile strength was 520 MPa, the yield stress was 358 MPa, and the elongation was 16.6% (Table 3).

Differences in properties in the transverse and longitudinal section were also confirmed by the authors of work [32] for WAAM 3D prints made of the Cu-Ni 70/30 alloy. Despite the much higher Ni content, the manufactured objects were characterized by lower mechanical properties by approximately 30% (UTS = 341–367 MPa, 112–114 HV0.1). The authors of work [18] for a standard CuNi10Fe1.8Mn alloy obtained an ultimate tensile strength of 235 MPa and a hardness of 86 HV (lower properties than the tested printed samples by about 52%) after the continuous casting process while high mechanical properties were obtained only after the cold working process with a degree of relative strain of 94%. In work [20], the authors proved that the use of an electromagnetic field in the continuous casting process results in a high degree of microstructure refinement and improvement in strength properties. For the cast CuNi10Fe1Mn alloy, the authors obtained an ultimate tensile strength of approximately 310 MPa (lower strength properties than the tested printed samples by approximately 37%). 

**Figure 12:**
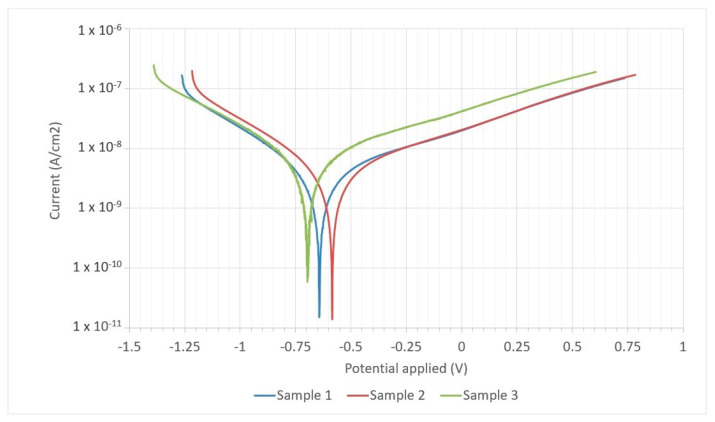
Polarization curves for tested samples from the 3D printed walls made of Cu-Ni 90/10 alloy.

The course of the polarization curves for the tested samples in 1 molar NaCl solution is shown in Figure 12, while the corrosion parameters determined on the basis of electrochemical tests are presented in Table 4.

A similar polarization curve was obtained in electrochemical measurements for three tested samples taken from different places on the walls. Compared to sample No. 1, the polarization curve for sample No. 2 was shifted towards the cathode current, while for sample No. 3, the polarization curve was shifted towards the plate current side. The polarization curves show clear Tafel sections, the extrapolation of which allows us to determine the value of the corrosion current density I_corr_. The average value of this parameter is approximately 3.6 × 10^−9^ A/cm^2^. The abscissa of the intersection point of the extrapolated lines allows for determining the value of the corrosion potential E_corr_. The average value of the corrosion potential calculated based on the obtained data is approximately −689 mV, while the average polarization resistance is approximately 2.6 × 10^7^ Ω. The average corrosion rate determined based on the formula from the ASTM G59-97 standard and electrochemical measurement data is approximately 7.4 × 10^−5^ mm/year. Based on the data obtained regarding the corrosion rate, the analyzed material can be classified based on the literature data [34] as low corrosion (below 0.025 mm/year) whereby the material is practically non-corrosive or the corrosion is very slow. Most works in the literature focused on the study of the corrosion properties of Cu-Ni 90/10 alloys [22,23,26,27,31,32] presenting electrochemical measurements carried out in the environment of a NaCl solution with a concentration of 1 to 3.5% or seawater solutions with additional chemical components [21,24,25,28]; therefore, it is difficult to directly compare the obtained results.

Further deeper studies of the microstructure are planned to determine the strengthening mechanism responsible for the high strength properties of 3D printed WAAM walls in relation to cast materials. Research is also carried out on the process of producing larger 3D objects using WAAM technology from the Cu-Ni 90/10 alloy of high structural and surface quality, as well as research on their mechanical and corrosion properties.

## 4. Conclusions

Based on the research conducted, the following conclusions and observations were formulated:

1. The developed WAAM 3D printing parameters allowed the creation of objects characterized by good quality and high shape regularity and are input data for determining the parameters of printing larger spatial objects made of the Cu-Ni 90/10 alloy using the WAAM technology;
2. The obtained walls are characterized by macro- and microstructures with large columnar grains oriented in the direction of heat dissipation to the baseplate and numerous precipitations of Ni-Ti phase particles, especially at the grain boundaries;
3. The produced walls are characterized by average hardness in the range of 138–160 HV10, ultimate tensile strength in the range of 495–520 MPa, conventional yield strength of 342–358 MPa, and elongation of 16.6–17.9%. The differences in the obtained mechanical properties in the longitudinal and transverse sections result from the structure of the oriented macrostructure and microstructure;
4. The Cu-Ni 90/10 alloy produced by WAAM 3D printing after electrochemical measurements in a 1 molar NaCl environment is characterized by an average value of corrosion potential of −689 mV, a corrosion current density of 3.6 × 10^−9^ A/cm^2^, and a resistance of 2.6 × 10^7^ Ω. The average corrosion rate is approximately 7.4 × 10^−5^ mm/year and this result is below 0.025 mm/year, which indicates that the material is practically non-corrosive or corrosion occurs very slowly;
5. The three-dimensional printed objects made of the Cu-Ni 90/10 alloy using commercial welding wire with the addition of Ti are characterized by higher strength properties above 30% compared to cast materials, which opens the possibility of using this alloy to produce objects with more complex shapes and for applications in corrosive environments.

## Figures and Tables

**Table 1:** Chemical composition of a commercial welding wire with a diameter of 1 mm made of Cu-Ni 90/10 alloy.

Element Content, %wt.					
Cu	Ni	Fe	Mn	Ti	Other
87.22	10.95	0.7	0.7	0.4	0.03

**Table 2:** Parameters of the 3D CMT-WAAM process used to produce spatial objects from the Cu-Ni 90/10 alloy.

Current, A	Voltage, V	Wire Feed Speed, m/min	Final Current, %	End Time, s
133	18.4	7	70	0.3

**Table 3:** Results of the static tensile test for samples from the transverse and longitudinal section of the Cu-Ni 90/10 alloy 3D wall.

Cross-Section	UTS, MPa	YS, MPa	A_50_, %
Transverse	495	342	17.9
Longitudinal	520	358	16.6

**Table 4:** Corrosion test results for samples from the longitudinal section of the Cu-Ni 90/10 alloy 3D wall determined by electrochemical measurements.

**Parameter**	**Sample 1**	**Sample 2**	**Sample 3**	**Average**
I_corr_, [A/cm^2^]	1.9 × 10^−9^	3.3 × 10^−9^	5.6 × 10^−9^	3.6 × 10^−9^ ± 1.53 × 10^−9^
E_corr, calc_ [mV]	−696	−622	−751	−689 ± 52.85
R_p_ [Ω]	3.4 × 10^7^	2.7 × 10^7^	1.8 × 10^7^	2.6 × 10^7^ ± 6.55 × 10^6^
Corrosion rate [mm/year]	7.1 × 10^−5^	7.5 × 10^−5^	7.7 × 10^−5^	7.4 × 10^−5^ ± 2.49 × 10^−6^

## Data Availability

Data are contained within the article.

## References

[1] Treutler K., Wesling V. (2021). The Current State of Research of Wire Arc Additive Manufacturing (WAAM): A Review. Appl. Sci..

[2] Wire Arc Additive Manufacturing (WAAM) Explained. https://www.makerverse.ai/metal-3d-printing/wire-arc-additive-manufacturing-(waam)-explained.

[3] Li J.L.Z., Alkahari M.R., Rosli N.A.B., Hasan R., Sudin M.N., Ramli F.R. (2019). Review of Wire Arc Additive Manufacturing for 3D Metal Printing. Int. J. Autom. Technol..

[4] Sian I., Evans J.W., Jian Q., Yongpeng H., Shepherd P., Ding J. (2022). A review of WAAM for steel construction—Manufacturing, material and geometric properties, design, and future directions. Structures.

[5] Rodríguez-González P., Ruiz-Navas E.M., Gordo E. (2023). Wire Arc Additive Manufacturing (WAAM) for Aluminum-Lithium Alloys: A Review. Materials.

[6] Zidong L., Kaijie S., Xinghua Y. (2021). A review on wire and arc additive manufacturing of titanium alloy. J. Manuf. Process..

[7] Dhinakaran V., Ajith J., Fathima Yasin Fahmidha A., Jagadeesha T., Sathish T., Stalin B. (2020). Wire Arc Additive Manufacturing (WAAM) process of nickel based superalloys—A review. Mater. Today Proc..

[8] Wang Y., Su C., Konovalov S. (2021). Microstructure and Mechanical Properties of Cu-6.5%Al Alloy Deposited by Wire Arc Additive Manufacturing. Metallogr. Microstruct. Anal..

[9] Dharmendra C., Hadadzadeh A., Amirkhiz B.S., Janaki Ram G.D., Mohammadi M. (2019). Microstructural evolution and mechanical behavior of nickel aluminum bronze Cu-9Al-4Fe-4Ni-1Mn fabricated through wire-arc additive manufacturing. Addit. Manuf..

[10] Xu C., Peng Y., Chen L.Y., Zhang T.H., He S., Wang K.H. (2023). Corrosion behavior of wire-arc additive manufactured and as-cast Ni-Al bronze in 3.5 wt% NaCl solution. Corros. Sci..

[11] Dharmendra C., Gururaj K., Pradeep K.G., Mohammadi M. (2021). Characterization of κ-precipitates in wire-arc additive manufactured nickel aluminum bronze: A combined transmission Kikuchi diffraction and atom probe tomography study. Addit. Manuf..

[12] Chen W., Chen Y., Zhang T., Wen T., Yin Z., Feng X. (2020). Effect of Ultrasonic Vibration and Interpass Temperature on Microstructure and Mechanical Properties of Cu-8Al-2Ni-2Fe-2Mn Alloy Fabricated by Wire Arc Additive Manufacturing. Metals.

[13] Govindaraj R.B., Junghans E., Andersen I., Lim Y.K., Lindström P. (2021). Additive manufactured marine component—Ni Al bronze propeller. Procedia Struct. Integr..

[14] Diao Z., Yang F., Xiong T., Chen L., Wu Y., Rong M. (2023). Microstructure and properties of CuCrZr alloy fabricated by wire arc additive manufacturing. Mater. Lett..

[15] Kazmi K.H., Sharma S.K., Das A.K., Mandal A., Shukla A. (2023). Development of Wire Arc Additive Manufactured Cu-Si Alloy: Study of Microstructure and Wear Behavior. J. Mater. Eng. Perform..

[16] Yugang M., Chunwang L., Yuyang Z., Yifan W., Ji L., Ziran W., Benshun Z. (2022). Material properties of gradient copper-nickel alloy fabricated by wire arc additive manufacturing base on bypass-current PAW. J. Manuf. Process..

[17] Copper Development Associciation The Aplication of Copper—Nickel Alloys in Marine Systems, Copper-Nickel Alloys: Properties, Processing, Application, Booklet, German Copper Institute (DKI), English Translation. https://www.copper.org/applications/marine/cuni/properties/DKI_booklet.html.

[18] Yanbin J., Xiaodong M., Yu L., Xinhua L., Yihan W., Jianxin X. (2019). Microstructure and mechanical property evolutions of CuNi10Fe1.8Mn1 alloy tube produced by HCCM horizontal continuous casting during drawing and its deformation mechanism. J. Alloys Compd..

[19] Taher A.M. (2016). Effect of Alloying Elements on the Hardness Property of 90% Copper-10% Nickel Alloy. Mater. Sci. Forum.

[20] Zhiming Y., Xintao L., Kai Q., Zhiqiang C., Xiaoli Z., Tingju L. (2009). Study on horizontal electromagnetic continuous casting of CuNi10Fe1Mn alloy hollow billets. Mater. Des..

[21] Parvizi M.S., Aladjem A., Castle J.E. (1988). Behaviour of 90–10 cupronickel in sea water. Int. Mater. Rev..

[22] Wu L., Ma A., Zhang L., Li G., Hu L., Wang Z., Zheng Y. (2023). Erosion–Corrosion Behavior of 90/10 and 70/30 Copper-Nickel Tubes in 1 wt% NaCl Solution. Metals.

[23] Zarebidaki A., Mofidi S.H.H., Bahri F.I. (2022). Effect of 2-mercaptobenzothiazole on the corrosion inhibition of Cu–10Ni alloy in 3 wt% NaCl solution. J. Appl. Electrochem..

[24] Taher A.M., Jarjoura G., Kipouros G.J. (2011). Effect of iron as alloying element on electrochemical behaviour of 90∶10 Cu–Ni alloy. Can. Metall. Q..

[25] Kear G., Barker B.D., Stokes K.R., Walsh F.C. (2007). Electrochemistry of non-aged 90–10 copper-nickel alloy (UNS C70610) as a function of fluid flow: Part 1: Cathodic and anodic characteristics. Electrochim. Acta.

[26] Kear G., Barker B., Stokes K., Walsh F.C. (2004). Electrochemical Corrosion Behaviour of 90—10 Cu—Ni Alloy in Chloride-Based Electrolytes. J. Appl. Electrochem..

[27] Shao G., Gao Y., Wu J., Liu P., Zhang K., Li W., Ma F., Zhou H., Chen X. (2022). Effect of Fe/Mn content on mechanical and corrosion properties of 90/10 copper-nickel alloy. Mater. Corros..

[28] Ying S., Liang L., Wenchang Y., Guangzhe C., Yong G. (2023). Investigation of corrosion behavior and film formation on 90Cu-10Ni alloys immersed in simulated seawater. Int. J. Electrochem. Sci..

[29] Chung S.W., Yun T.N., Joong Kang C.Y. (2018). Microstructure and Mechanical Properties in the Friction Stir Welded C70600 Alloy. J. Weld. Join..

[30] Hsissou R., Benzidia B., Hajjaji N., Elharfi A. (2018). Elaboration and Electrochemical Studies of the Coating Behavior of a New Nanofunctional Epoxy Polymer on E24 Steel in 3.5% NaCl. Port. Electrochim. Acta.

[31] Mohd Z.M., Mohd Y., Che I., Nik N. (2017). Cu-Ni Alloys Coatings For Corrosion Protection On Mild Steel In 0.5 M NaCl Solution. Sci. Lett..

[32] Guo C., Kang T., Wu S., Ying M., Liu W.M., Chen F. (2021). Microstructure, mechanical, and corrosion resistance of copper nickel alloy fabricated by wire-arc additive manufacturing. MRS Commun..

[33] (2014). Standard Test Method for Conducting Potentiodynamic Polarization Resistance Measurements.

[34] Mainier F., Coelho A., Barros E. (2019). Corrosivity Evaluation of Copper-Nickel Alloy (90/10) in Pumps Used in Offshore Platforms for Seawater Pumping. ETASR.

